# 3DG Functionalized Magnetic Solid Phase Extraction Materials for the Efficient Enrichment of Hexamethylenetetramine in Vermicelli

**DOI:** 10.3390/molecules27051548

**Published:** 2022-02-25

**Authors:** Min Liu, Chen Zhu, Xiaoyan Li, Fang Wang

**Affiliations:** 1 Key Laboratory of Guangxi Colleges and Universities for Food Safety and Pharmaceutical Analytical Chemistry, Guangxi Key Laboratory of Chemistry and Engineering of Forest Products, School of Chemistry and Chemical Engineering, Guangxi University for Nationalities, Nanning 530008, China; 2016081792440@stu.gxun.edu.cn; 2 Guangxi Key Laboratory of Utilization of Microbial and Botanical Resources, School of Marine Sciences and Biotechnology, Guangxi University for Nationalities, Nanning 530008, China; gxpsmm@gxun.edu.cn

**Keywords:** magnetic solid phase extraction, 3D graphene, “thiol-ene” click chemistry, high-performance liquid chromatography, hexamethylenetetramine

## Abstract

Solid phase extraction (SPE) is regarded as the most effective purification method for complex matrix samples owing to its simplicity of operator, time-saving, high accuracy, and environmental friendliness. SPE technology is still affected by the high cost of commercial SPE columns and poor adsorption selectivity. Hence, the development of low-cost and highly selective adsorbents is quite challenging and demanding in SPE. In this study, a novel 3DG functionalized magnetic solid phase extraction materials was prepared based on “thiol-ene” click chemistry. The structure, morphology, thermal stability, and magnetic properties of the magnetic composites were studied by Fourier transform infrared spectroscopy (FT-IR), scanning electron microscopy (SEM), energy dispersive spectroscopy (EDS), thermogravimetric analysis (TGA), and vibrating sample magnetometer (VSM). Then the adsorption performance of composite was determined by static adsorption experiments, which showed fast binding kinetics (100 min) and good adsorption performance (*Q_e_* *=* 65.34 mg/g). Moreover, these magnetic nanoparticles were used as adsorbents for magnetic solid phase extraction (MSPE) and coupled with high-performance liquid chromatography (HPLC) for separation and detection of illegally added hexamethylenetetramine in vermicelli. As for practical application, the recoveries for the spiked samples in the concentration range of 8–40 μg/g were between 83.24–92.69%, and the RSD was between 0.20–2.07%.

## 1. Introduction

Hexamethylenetetramine (HMT) is an industrially important raw material and common drug, often used as a curing agent for resins and for the treatment of livestock diseases [1,2,3]. It is sometimes illegally added to food, such as in bean vermicelli and dried beancurd sticks, to increase profits. HMT not only improves the taste of food, but also releases formaldehyde and ammonia under acidic conditions [4], and hence improves the whitening and anti-corrosion properties [5]. However, formaldehyde released by HMT are carcinogenic and toxic, endangering human health [6]. Therefore, the National Health Commission of China promulgated a list of non-edible and food additives that may be added illegally to food; the list includes 151 illegal or regulated substances, such as sulfa antibiotics and HMT, that have been found in food and feed over the past few years [7]. Other countries such as the United States and Australia have limited or forbidden the addition of HMT in food manufacturing [8,9]. There is also an urgent need to develop a proper separation and detection method to crack down on the illegal addition of HMT in food.

Presently, the commonly used methods for detection of HMT include spectrophotometry [10], chromatography [11], Raman spectroscopy [12], and high-performance liquid chromatography/tandem mass spectrometry method (HPLC-MS/MS) [13]. However, detection of trace components directly from a complex matrix by these methods is difficult. Hence, these methods of analysis require suitable sample pretreatments, such as the QuEChERS method [13] and SPE method [14,15]. These methods are often time-consuming, complicated, and usually coupled with expensive mass spectrometric techniques requiring isotopic labeling. Therefore, in order to properly develop these detection techniques, it is necessary to explore a more economical and practical sample pretreatment technique for the separation and enrichment of HMT in complex food.

Magnetic solid phase extraction (MSPE) is an important sample pretreatment technique [16], which is developing rapidly and has been widely applied for testing of food, medicine, and the environment. Novel 3DG was chosen for the fabrication of magnetic functional materials employing “click chemistry”. 3DG is a kind of graphene material with a specific three-dimensional spatial micro/nanostructure structure assembled from graphene sheets [17]. This structure combines the intrinsic properties of graphene with the three-dimensional porous structure having a larger specific surface area and a greater number of active sites [18]. Therefore, it has been widely used in composite materials recently [19,20]. “Click chemistry” is a new synthetic approach for drugs and polymer proposed by Sharpless, which has characteristics of high efficiency, reliability, selectivity, and non-metal catalysis [21]. The “thiol-ene” click reaction is one of the simplest reactions in click chemistry because of its high reaction efficiency extensive applications [22].

In this study, a novel composite for MSPE was developed and coupled with HPLC to efficiently extract and determine the amount of HMT, in order to overcome the shortcomings of the traditional methods. The performance of this novel composite was improved due to the 3D graphene structure, which endowed it with a more specific surface area, ease of modification, and functionalization [23,24]. Meanwhile, “thiol-ene” click reaction has made the synthesis process of composite more efficient and environmentally friendly [21,22]. Then, the nanocomposite was characterized and the thermodynamic and kinetic studies of adsorption were carried out. The obtained composite was applied as magnetic solid phase extraction material for separation and enrichment of HMT, and then combined with HPLC to detect HMT in actual samples.

## 2. Results and Discussion

**Scheme 1:**
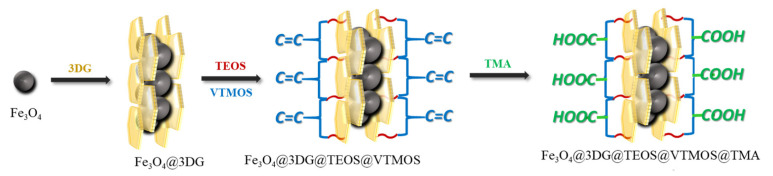
Graphic illustration preparation of Fe_3_O_4_@3DG@VTMOS@TMA.

In this study, Fe_3_O_4_ nanoparticles were synthesized by the chemical coprecipitation method. Then, 3DG was used to wrap up the surface of the Fe_3_O_4_ nanoparticles to afford Fe_3_O_4_@3DG with a high specific surface area. The surface fixed vinyl groups of silanized Fe_3_O_4_@3DG@VTMOS coated on Fe_3_O_4_@3DG were prepared by the sol-gel method; this surface modification effectively improved the dispersion magnetite nanoparticles in liquid media and enhanced the stability of the compound. Fe_3_O_4_@3DG@VTMOS were further reacted with TMA to introduce sulfhydryl groups through “thiol-ene” click chemistry under milder conditions to obtain Fe_3_O_4_@3DG@VTMOS@TMA. The methodology of preparation of Fe_3_O_4_@3DG@VTMOS@TMA is presented in Scheme 1.

### 2.1. Characterizations

Fe_3_O_4_, Fe_3_O_4_@3DG, Fe_3_O_4_@3DG@VTMOS, and Fe_3_O_4_@3DG@VTMOS@TMA composites were characterized by SEM, EDS, FT-IR, TGA, and VSM.

**Figure 1:**
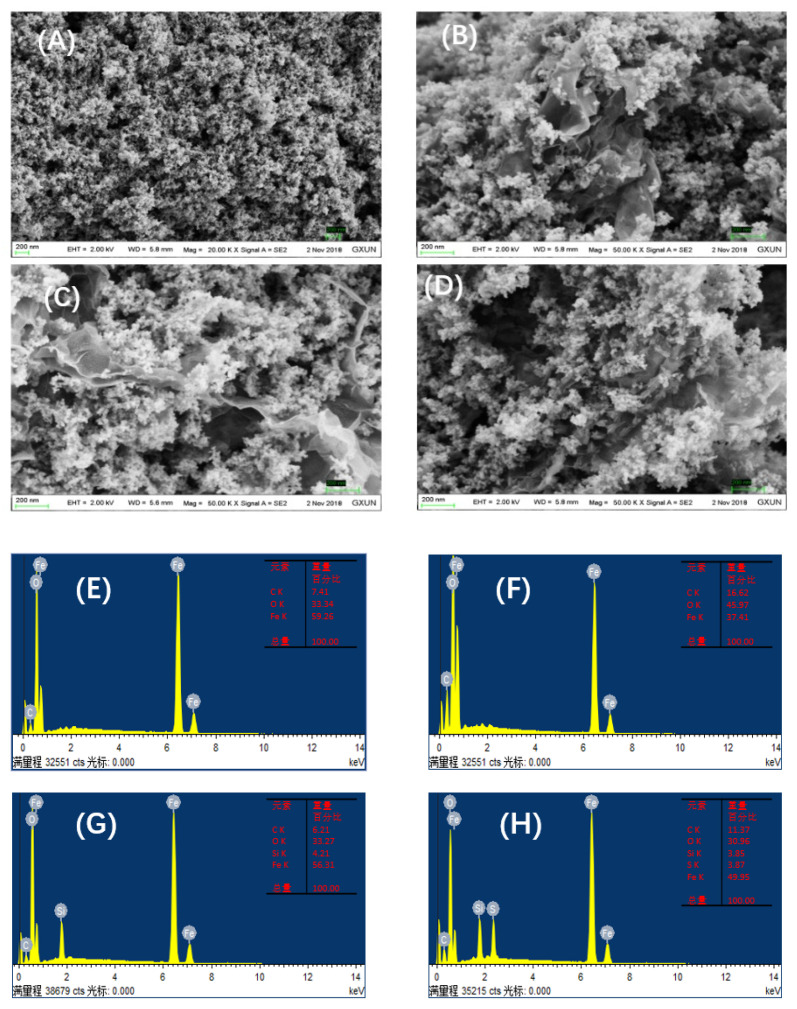
SEM and EDS images of Fe_3_O_4_ (**A**,**E**), Fe_3_O_4_@3DG (**B**,**F**), Fe_3_O_4_@3DG@VTMOS (**C**,**G**), and Fe_3_O_4_@3DG@VTMOS@TMA (**D**,**H**).

The morphologies and elemental analysis of Fe_3_O_4_, Fe_3_O_4_@3DG, Fe_3_O_4_@3DG@VTMOS, and Fe_3_O_4_@3DG@VTMOS@TMA were examined by SEM and EDS spectra (Figure 1A–H). Figure 1A showed that Fe_3_O_4_ nanoparticles were regularly spherical in shape with a uniform size distribution, The average size of the Fe3O4 nanoparticles was found to be in the range of 20–30 nm by Nano Measurer software. As seen in Figure 1E, Fe_3_O_4_ nanoparticles were composed of the elements iron and oxygen. Trace carbon elements may be introduced by sample preparation with the electric conductive tapes. In Figure 1B, the surface of Fe_3_O_4_@3DG nanoparticles has a fluffy fold structure, wherein the specific surface area of the composite was increased. From Figure 1F, the weight percentage of carbon elements increased compared to Fe_3_O_4_ nanoparticles. It proves that 3DG was loaded on the surface of Fe3O4 successfully and homogeneously. Figure 1C,D showed that the particle sizes of Fe_3_O_4_@3DG@VTMOS and Fe_3_O_4_@3DG@VTMOS@TMA nanoparticles became bigger with the increase of surface modification layers of Fe_3_O_4_ nanoparticles. The presence of silicon and sulfur suggested that VTMOS and TMA were sufficiently polymerized on the surface of the composite (Figure 1G,H). Moreover, the appearance of 3DG is still visible on the surface of the composite also indicates the successful polymerization of the composite.

**Figure 2:**
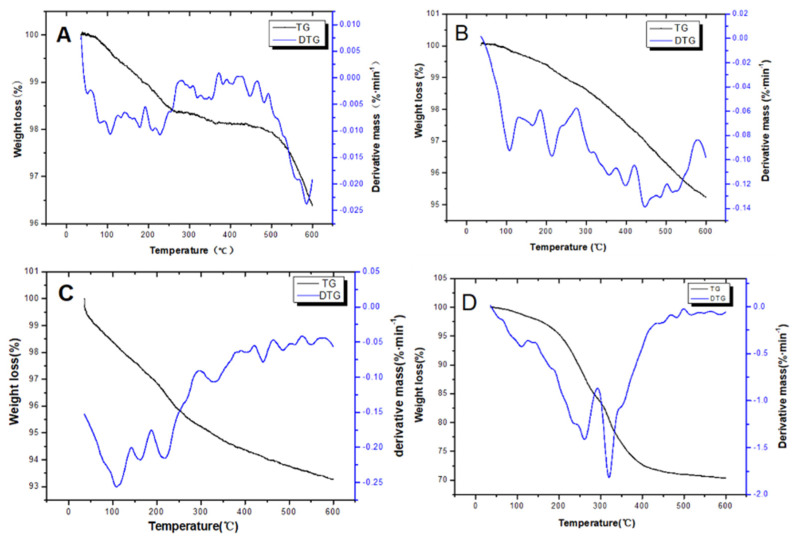
The TG and DTG curves of Fe_3_O_4_ (**A**), Fe_3_O_4_@3DG (**B**), Fe_3_O_4_@3DG@VTMOS (**C**), and Fe_3_O_4_@3DG@VTMOS@TMA (**D**).

The TG and DTG analysis of Fe_3_O_4_ (Figure 2A), Fe_3_O_4_@3DG (Figure 2B), Fe_3_O_4_@3DG@VTMOS (Figure 2C), Fe_3_O_4_@3DG@VTMOS@TMA (Figure 2D) are shown in Figure 2. The weight losses of Fe_3_O_4_, Fe_3_O_4_@3DG, Fe_3_O_4_@3DG@VTMOS (Figure 2C), Fe_3_O_4_@3DG@VTMOS@TMA (Figure 2D) were 1.60, 1.20, 3.16, 4.65% from 35 °C to 200 °C, respectively, the weight loss was caused by the evaporation of residual solvents. As seen in Figure 2A, there is no obvious change in weight loss (0.4%) from 200 to 480 °C. The weight loss was about 1.64% after 480 °C, which was mainly attributed to Fe_3_O_4_ loses weight [25]. The TG and DTG curves of Fe_3_O_4_@3DG and Fe_3_O_4_@3DG@VTMOS exhibited weight loss peaks at 448.26 °C and 331.84 °C, respectively. The weight loss peak was caused by the decomposition of 3DG and sialylation coated on the surface of Fe_3_O_4._ The DTG curves of Fe_3_O_4_@3DG@VTMOS@TMA in Figure 2D contained two obvious weight loss peaks. The TG curves ranged from 200 °C to 280 °C. The weight losses were 10.05%. The weight loss peak at 262.28 °C was caused by the breaking of the bond between Fe_3_0_4_ and TMA, from 280 °C to 400 °C. The weight loss was 13.63%, with the weight loss peak at 319.92 °C. The other weight loss peak was caused by the burning of depolymerization between TMA units [26]. The weight losses of Fe_3_O_4_@3DG, Fe_3_O_4_@3DG@VTMOS, and Fe_3_O_4_@3DG@VTMOS composites were 5.0, 7.0, and 29.0%, respectively, between 35–600 °C.; this makes it evident that as Fe_3_O_4_ was modified to Fe_3_O_4_@3DG@VTMOS, it became more and more prone to thermal degradation.

**Figure 3:**
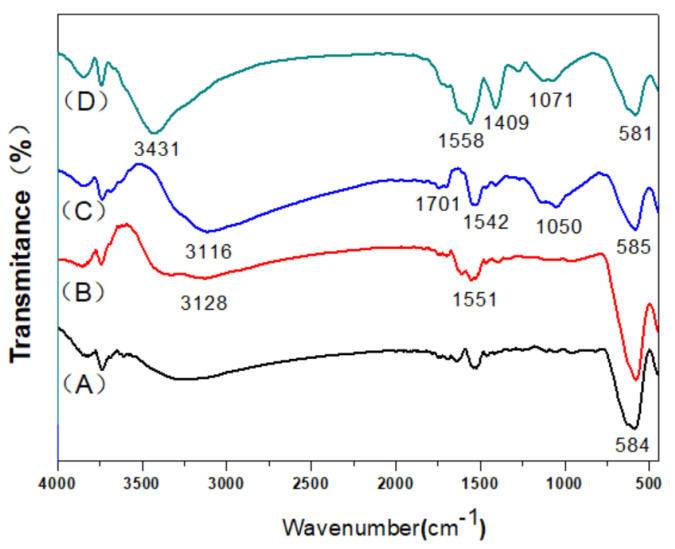
The FT-IR spectra of Fe_3_O_4_ (**A**), Fe_3_O_4_@3DG (**B**), Fe_3_O_4_@3DG@VTMOS (**C**), and Fe_3_O_4_@3DG@VTMOS@TMA (**D**).

FT-IR determines the structure of molecules or functional groups with infrared radiant energy. The FT-IR spectra of Fe_3_O_4_ (Figure 3A), Fe_3_O_4_@3DG (Figure 3B), Fe_3_O_4_@3DG@VTMOS (Figure 3C), and Fe_3_O_4_@3DG@VTMOS@TMA (Figure 3D) are shown in Figure 3. The peak at 580 cm^−1^ in the spectrum of Fe_3_O_4_ could be attributed to the Fe-O stretching vibrations (Figure 3A) [27]. In the spectrum of Fe_3_O_4_@3DG (Figure 3B), peaks at 1551 cm^−1^ and 3128 cm^−1^ could be ascribed to C=C stretching and C-H stretching vibrations of CH=CH_2_ group of 3DG, respectively [28]. This indicated that 3DG was successfully coated on the surface of Fe_3_O_4_ particles. Peaks at 1542 cm^−1^, 1701 cm^−1^, and 3116 cm^−1^ in the spectrum of Fe_3_O_4_@3DG@VTMOS (Figure 3C) were assigned to C=C, C=O, and C-H of CH=CH_2_, respectively. Moreover, stretching vibrations of Si-O-Si groups were observed at 1050 cm^−1^ [29]. Furthermore, peaks at 1409 cm^−1^ and 3431 cm^−1^ in the spectrum of Fe_3_O_4_@3DG@VTMOS@TMA, corresponding to C-C and -OH stretching vibrations of carboxyl groups (Figure 3D), confirmed the formation of the product.

**Figure 4:**
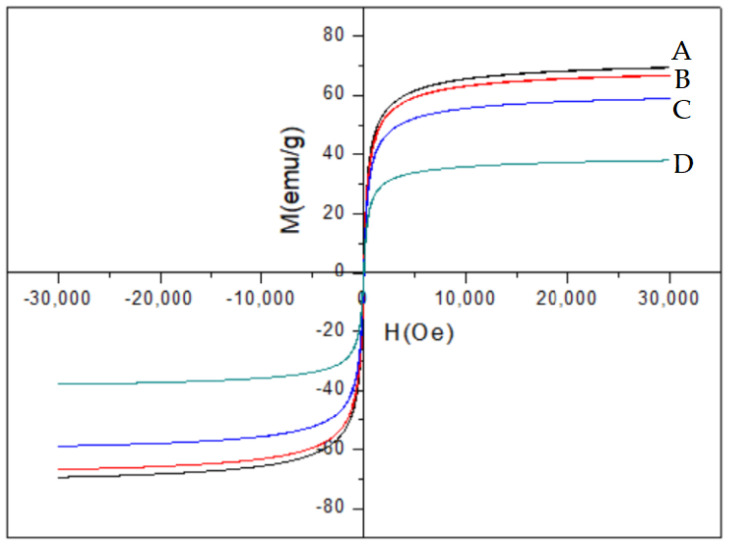
Magnetization curves of Fe_3_O_4_ (**A**), Fe_3_O_4_@3DG (**B**), Fe_3_O_4_@3DG@VTMOS (**C**), and Fe_3_O_4_@3DG@VTMOS@TMA (**D**).

The VSM curves of Fe_3_O_4_ (Figure 4A), Fe_3_O_4_@3DG (Figure 4B), Fe_3_O_4_@3DG@VTMOS (Figure 4C), and Fe_3_O_4_@3DG@VTMOS@TMA (Figure 4D) are shown in Figure 4. Results showed that all the composites possessed superparamagnetism and their saturation magnetization values were 69.33, 66.65, 58.86, and 38.04 emu/g, respectively. The magnetization values decreased as the Fe_3_O_4_ surface was modified. It was also clear that the Fe_3_O_4_@3DG@VTMOS@TMA could be simply separated using an external magnetic field.

### 2.2. Study of Adsorption Properties

**Figure 5:**
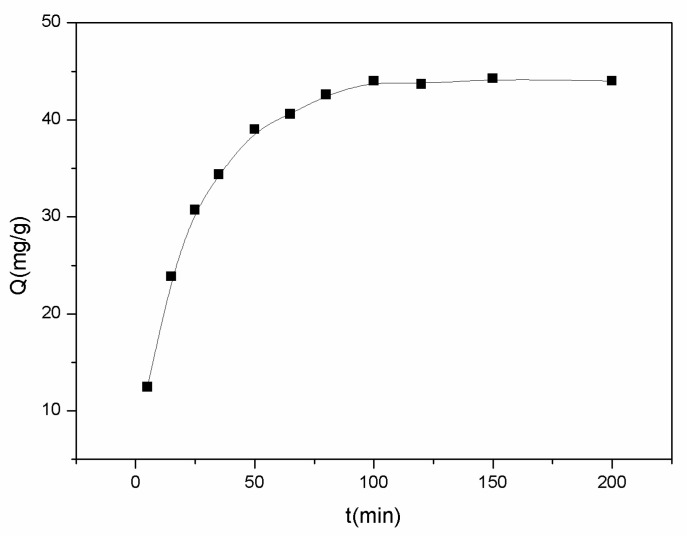
The adsorption kinetics of Fe_3_O_4_@3DG@VTMOS@TMA.

The kinetics and thermodynamics of adsorption of Fe_3_O_4_@3DG@VTMOS@TMA on HMT were investigated experimentally. Figure 5 shows the adsorption capacities of Fe_3_O_4_@3DG@VTMOS@TMA for HMT with a concentration of 1.0 mg/mL. It can be seen that the adsorption capacities of Fe_3_O_4_@3DG@VTMOS@TMA for HMT increased with time and remained basically unaltered from 100 to 300 min, and hence it can be inferred that the adsorption equilibrium was attained at 100 min.

To further study the kinetic mechanisms of the adsorption process, the pseudo-first-order and pseudo-second-order kinetic models were applied to fit the kinetic data according to the following equations [30,31,32]:

(1) $$\ln Q_{e}-Q_{t}=\ln Q_{e\;}-k_{1}t$$

(2) $$t/Q_{t}=t/Q_{e}+1/k_{2}Q_{e}^{2}$$

where *k*_1_ and *k*_2_ are the rate constants of pseudo-first-order and pseudo-second-order kinetic models; *Q_e_* and *Q_t_* are the adsorption capacities (mg/g) at equilibrium and at time *t* (min); *Q_e_* and *k* can be calculated from the plots of ln(*Q_e_* − *Q_t_*) versus *t* and *t/Q_t_* versus *t*, respectively.

From the data in Table 1, the adsorption kinetics of Fe_3_O_4_@3DG@VTMOS@TMA conformed well to the pseudo-second-order model (*R*^2^ = 0.9982), which could be expressed as $t/Q_{t}=0.02094t+0.2617$.

**Figure 6:**
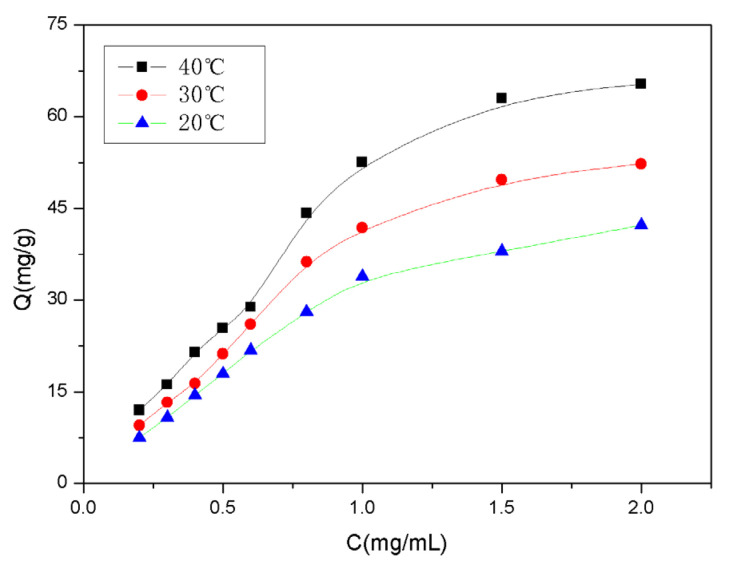
Adsorption isotherms of Fe_3_O_4_@3DG@VTMOS@TMA.

Thermodynamic parameters of adsorption of HMT on Fe_3_O_4_@3DG@VTMOS@TMA were evaluated from the adsorptivities. This data was used to plot the adsorption isotherm of Fe_3_O_4_@3DG@VTMOS@TMA for different initial concentrations range of HMT from 0.2 to 2.0 mg/mL. As shown in Figure 6, the amount of HMT bound to Fe_3_O_4_@3DG@VTMOS@TMA increased rapidly with an increase in initial concentration up to 1.5 mg/mL. Binding saturation was attained above 1.5 mg/mL concentration between 20–40 °C. Furthermore, adsorptivity increased gradually with increased in adsorption temperature.

Thermodynamic parameters such as enthalpy change (Δ*H^θ^*), entropy change (Δ*S^θ^*), and the Gibb’s free energy change (Δ*G^θ^*) for Fe_3_O_4_@3DG@VTMOS@TMA were calculated from the following equations [33]:

(3) $$\Delta G^{\theta }=-RT\ln K_{d}$$

(4) $$\ln K_{d}=-\Delta H^{\theta }/RT+\Delta S^{\theta }/R$$

(5) $$K_{d}=C_{p}/C_{s}$$

where *R* is the gas constant (8.314 J/mol·K) and *T* is the reaction temperature (*K*).

Plotting ln*K_d_* versus *1/T*, the fitting equation is $\ln K_{d}=-2260.01/T+11.31$ (*R*^2^ = 0.9972). Δ*H^θ^* and Δ*S^θ^* were obtained from the plots. The thermodynamic parameters for the adsorption of HMT on Fe_3_O_4_@3DG@VTMOS@TMA, shown in Table 2, were calculated from the intercept and slope of the equation.

The negative values of Δ*G^θ^* suggested that the spontaneity of the adsorption process increased with increasing temperature. Additionally, the positive values of Δ*H^θ^* indicated that the adsorption process was endothermic, whereas the positive Δ*S^θ^* value suggested that the randomness increased in the adsorption system.

In order to further investigate the adsorption performance of Fe_3_O_4_@3DG@VTMOS@TMA for HMT, the adsorption isotherms and parameters were described by the Langmuir equation [34] and Freundlich equation [35], respectively:

(6) $$1/Q_{e}=1/Q_{max}K_{L}C_{e}+1/Q_{max}$$

where *Q_max_* is the maximum adsorptivity at adsorption equilibrium (mg/g); *K_L_* represents the equilibrium adsorption constant.

(7) $$\ln Q_{e}=\ln C_{e}/n+\ln K_{F}$$

where *K_F_* represents the adsorption capacity and *n* is the adsorption intensity. Equilibrium adsorption data were modeled by plotting 1*/Q_e_* versus 1*/C_e_* and ln*Q_e_* versus ln*C_e_* to fit the Langmuir and Freundlich equations, respectively.

As shown in Table 3, the adsorption of HMT on Fe_3_O_4_@3DG@VTMOS@TMA was well consistent with the Langmuir adsorption model (0.9891 < *R*^2^ < 0.9942), which suggested a monolayer of adsorption. Besides, *Q_max_* and *K_L_* increased with an increase in temperature. The values of *Q_max_* obtained by the Langmuir equation were higher than the actual measured values, indicating that the synthesis conditions can be continuously optimized to improve the adsorptivity of the Fe_3_O_4_@3DG@VTMOS@TMA. The adsorption of HMT on Fe_3_O_4_@3DG@VTMOS@TMA was mainly controlled by chemical adsorption in nature.

### 2.3. Validation of Method

The method was validated by plotting the standard calibration curve using solutions of HMT in the concentration range from 1.0 to 10.0 µg/mL. The linear regression equations for HMT was expressed as S=51.7850C−0.3372 (*R*^2^ = 0.9997), which was obtained from the plot of peak areas versus mass concentration for HMT. The limit of detection (LOD, S/N = 3) and the limit of quantification (LOQ, S/N = 10) measured for HMT were 0.25 and 0.60 µg/mL, respectively.

### 2.4. Analysis of the Real Samples

In order to validate the practical application of the MSPE-HPLC method, spike and recovery experiments for the analysis of HMT in vermicelli samples with spike levels of 8, 20, and 40 µg/g (Table 4) were carried out. These samples were randomly purchased from different manufacturers and markets. No HMT was detected in these samples above the levels examined. The recovery and the RSD were in the ranges of 83.24–92.69% and 0.20–2.07%, respectively.

**Figure 7:**
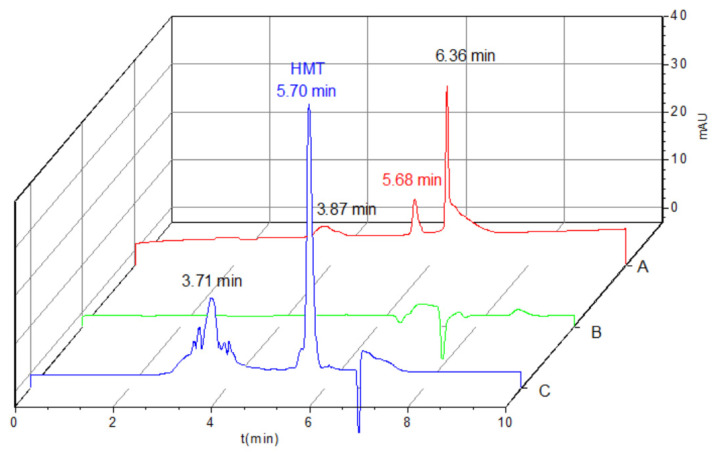
Chromatograms of spiked vermicelli sample before MSPE (**A**), of raffinate (**B**), and after MSPE (**C**) (40 µg/g).

Figure 7 shows the chromatograms of a spiked vermicelli sample before MSPE (Figure 7A), of raffinate (Figure 7B), and after MSPE (Figure 7C) (40 µg/g). After MSPE of spiked vermicelli samples using Fe_3_O_4_@3DG@VTMOS@TMA, the peak of HMT (5.70 min) was more intense than that before MSPE (5.68 min) without interference. These results indicate that the novel 3DG synergistic composite can not only adsorb the illegally added HMT, but also enrich it in the vermicelli samples.

Compared with other methods (Table 5), although the recovery of the proposed method was lower, the LOD and LOQ were better. Furthermore, the RSD values were also significantly smaller. Results showed that the MSPE-HPLC method established in this experiment had good accuracy and precision.

## 3. Materials and Methods

### 3.1. Reagents

All chemicals used were of analytical reagent grade unless otherwise specified. Hexamethylenetetramine (HMT) and mercaptosuccinic acid (TMA) were obtained from Sigma-Aldrich (St. Louis, MO, USA). Vinyltrimethoxysilane (VTMOS) was purchased from Fluorochem (Derbyshire, UK). Tetraethoxysilane (TEOS) was obtained from Tianjin Kemiou Chemical Reagent (Tianjin, China). Iron dichloride (FeCl_2_·4H_2_O) and iron chloride (FeCl_3_·6H_2_O) were acquired from Chron Chemicals (Chengdu, Sichuan, China). 2,2′-Azobisisobutyronitrile (AIBN) was obtained from J&K Chemical (Beijing, China). HPLC-grade acetonitrile was purchased from Fisher Scientific (Fairlawn, NJ, USA). Deionized water was obtained using a Milli-Q water purification system (Merck, Saint-Quentin, France).

### 3.2. Apparatus

Scanning electron microscopy (SEM) was performed using a SUPRA 55 Sapphire field emission scanning electron microscope equipped with OXFORD X-MaxN51-XMX1004 (Carl Zeiss, Jena, Germany). The samples were prepared by spreading the powder onto double sided carbon tape, mounted onto microscope holder, the gold was supported on the surface of the material by the sputter coating of the sample preparation technique. SEM images were captured at 20.00–50.00 K magnification and varying voltages of 2.00 kV. The Fourier-transform infrared (FT-IR) spectra were recorded on a Magna IR550 (II) type spectrophotometer (Nicolet, Waltham, MA, USA) using a KBr disc with a scanning range of 4000 to 400 cm^−1^ and wavenumber resolution was 4cm^−1^. Thermogravimetric analysis (TGA) was performed by the TG209F1 thermogravimetric analyzer (Netzsch, Selb, Germany) under nitrogen atmosphere in the temperature range of ambient temperature to 600 °C, at a heating rate of 10 °C/min. Magnetic properties of the samples were determined using a vibrating sample magnetometer (VSM) of Quantum Design PPMS DynaCool model (San Diego, CA, USA). The ultraviolet–visible (UV-vis) absorption spectra were recorded using a UV-1800 spectrophotometer from Suzhou Shimadzu Corporation (Suzhou, Jiangsu, China).

### 3.3. Preparation of Fe_3_O_4_@3DG@VTMOS@TMA

Fe_3_O_4_ nanoparticles were prepared by the coprecipitation method. Graphene (GO) was synthesized by a modified Hummers method, after which the 3D graphene (3DG) was wrapped onto the surface of Fe_3_O_4_ to obtain black Fe_3_O_4_@3DG. A mixture of TEOS (2 mmol) and Fe_3_O_4_@3DG (300 mg) was dissolved in 50 mL of ethanol ultrasonically for 5 min then transferred to a three-necked flask. VTMOS (2 mmol) dissolved in 50 mL of ethanol was added dropwise to the above solution. Aqueous ammonia was used to adjust the pH to 10 and stirred at 55 °C for 3 h in a nitrogen atmosphere. After completion of the reaction, the product was washed thoroughly with deionized water till neutral pH value to obtain silanized Fe_3_O_4_@3DG@VTMOS, containing the vinyl groups. Solution of Fe_3_O_4_@3DG@VTMOS (300 mg) in ethanol/water (50 mL, 25:25, *v/v*) was transferred to a three-necked flask. Finally, TMA (3 mmol) and AIBN (0.1 g) dissolved in 50 mL of ethanol were added in turn. The reaction mixture was stirred for 3 h at 75 °C in a nitrogen atmosphere. After polymerization, the product was washed with ethanol. Thus, Fe_3_O_4_@3DG@VTMOS@TMA composite was obtained after drying overnight in a vacuum oven at 55 °C.

The reaction of thiols with enes was defined as a free radical polymerization with thiol–ene free-radical addition to electron-rich/electron-poor carbon–carbon double bonds under mild conditions [39]. The thiols-enes click reaction possesses the characteristics of a simple procedure, mild reaction conditions, and high purity product, and is popular in the surface modification of materials. In this study, vinyl functional groups of VTMOS were coated on the surface of Fe_3_O_4_@3DG by sol-gel method, then TMA grafted to the surface of Fe_3_O_4_@3DG@VTMOS by the thiols-enes click reaction. The material was prepared by this method with high yield and minimal side reactions.

### 3.4. Adsorption Experiments

Static adsorption tests were carried out to investigate the adsorptivity of the Fe_3_O_4_@3DG@VTMOS@TMA. In a typical procedure, Fe_3_O_4_@3DG@VTMOS@TMA (25 mg) was put into a wide-mouth bottle containing 10 mL of HMT solution. The mixture was incubated for a certain time. Then Fe_3_O_4_@3DG@VTMOS@TMA was separated from the above solution using an external magnetic field. The concentration of HMT in the supernatant was determined at 241 nm, and the adsorptivity was calculated by the following equation:

(8) $$Q_{e}=\left(C_{o}-C_{e}\right)V/w$$

where *Q_e_* (mg/g) is the binding quantity for the composite at equilibrium; *C_o_* and *C_e_* (mg/mL) are the initial and equilibrium solution concentrations of the solution, respectively; *V* (mL) is the volume of the solution and *w* (g) is the weight of the composite.

### 3.5. MSPE-HPLC Analysis of HMT in Vermicelli Samples

Random samples of vermicelli were purchased from local markets. Powder of commercially available vermicelli (5 g) and a certain volume of 1.0 mg/mL standard acetonitrile solution of HMT were mixed together to obtain spiked sample mixtures at the spiking level of 8–40 μg/g. The above sample mixtures were ultrasonically dispersed in an ice-water bath for 10 min and mixed well before centrifugation at 6000 rpm for 5 min. Then, the supernatant (15 mL) was separated and evaporated to dryness. The residue was extracted into trichloromethane (15 mL). Thereafter, accurately 10 mL of the above solution was added to Fe_3_O_4_@3DG@VTMOS@TMA (50 mg) and shaken at 40 °C for 100 min. After adsorption, Fe_3_O_4_@3DG@VTMOS@TMA was separated using an external magnetic field. Then, HMT was eluted with 3 × 1.0 mL of ammonia/acetonitrile (5:95, *v*/*v*) and the obtained eluent was evaporated to dryness under a stream of nitrogen. Ultimately, the residue was enriched, extracted with acetonitrile (1.0 mL), and filtered through a 0.22 μm nylon membrane for HPLC analysis. HPLC analysis was performed using an Agilent 1260 HPLC equipped with DAD detector. A Phenomenex Gemini C18 analytical column (250 mm × 4.6 mm, 5 μm particle size) was used for the separation of analytes with a mobile phase consisting of acetonitrile/water (5:95, *v*/*v*) as the mobile phase at a flow rate of 0.5 mL/min (35 °C). The injection volume was 20 μL, and the wavelength of the detector was monitored at 210 nm.

## 4. Conclusions

In this study, a novel 3DG synergistic composite, Fe_3_O_4_@3DG@VTMOS@TMA, was prepared employing “thiol-ene” click chemistry. The composite product had a fast adsorption rate and high adsorptivity for HMT. Moreover, the experimental results of recovery showed that the proposed MSPE-HPLC method could be successfully applied for the extraction and determination of HMT in vermicelli samples. Therefore, it is a promising method for the rapid separation and determination of illegally added HMT using Fe_3_O_4_@3DG@VTMOS@TMA coupled with HPLC.

## Figures and Tables

**Table 1:** Regression parameters of the pseudo-first-order and pseudo-second-order equations.

Kinetic Model	Adsorption Equation	*R* ^2^	*Q_e_* (mg/g)
pseudo-first-order	$\ln(Q_{e}-Q_{t})=-0.04590t+3.6310$	0.4693	37.75
pseudo-second-order	$t/Q_{t}=0.02094t+0.2617$	0.9982	47.76

**Table 2:** Thermodynamic parameters for adsorption of hexamethylenetetramine by Fe_3_O_4_@3DG@VTMOS@TMA.

Δ*H^θ^*	Δ*S^θ^*	Δ*G^θ^* (KJ/mol)			*R* ^2^
(KJ/mol)	(J/mol·K)	293.15 K	303.15 K	313.15 K	
18.79	90.03	−8.80	−9.38	−10.68	0.9972

**Table 3:** Regression of Langmuir and Freundlich isotherm equations.

Temperature (K)	Adsorption Isotherm			
	**Freundlich Isotherm Parameters**			
	**Regression Equations**	***Q_max_* (mg/g)**	***K* *_L_***	***R* ^2^**
293.15	1/*Q_e_* = 0.02520/*C_e_* + 0.006767	147.78	0.27	0.9942
303.15	1/*Q_e_* = 0.02026/*C_e_* + 0.006184	161.71	0.31	0.9903
313.15	1/*Q_e_* = 0.01602/*C_e_* + 0.005773	173.22	0.36	0.9891
	**Freundlich Isotherm Parameters**			
	**Regression Equations**	***K_F_* (mg/g)**	***n***	***R* ^2^**
293.15	ln*Q_e_* = 0.7794ln*C_e_* + 3.3809	29.40	1.28	0.9595
303.15	ln*Q_e_* = 0.7999ln*C_e_* + 3.5967	36.48	1.25	0.9606
313.15	ln*Q_e_* = 0.8019ln*C_e_* + 3.8060	44.97	1.25	0.9654

**Table 4:** Recoveries of HMT bound to composite in spiked vermicelli samples (*n* = 5).

Samples	Spiked Level					
Vermicelli	8 μg/g		20 μg/g		40 μg/g	
	**Recovery (%)**	**RSD (%)**	**Recovery (%)**	**RSD (%)**	**Recovery (%)**	**RSD (%)**
1	89.46	1.95	85.93	2.07	91.42	0.70
2	87.44	1.09	86.43	1.15	92.69	0.20
3	86.24	1.64	83.24	0.56	91.19	0.43

**Table 5:** Comparison of proposed MEPS-HPLC method to determine HMT with other analytical methods reported in the literature.

Method	LOD	LOQ	Spiked Level	Recovery	RSD	References
	(µg/mL)	(µg/mL)	(μg/g)	(%)	(%)	
HPLC	1	-	4–40	93.5–98.7	1.5–4.2	[36]
HPLC	5	10	10–100	91.6–100.9	0.9–8.7	[37]
SERS	0.5	-	0.5–5.0	-	0.30–3.83	[12]
LC-MS	0.6	1.0	25–200	20.9–106.9	-	[38]
MSPE-HPLC	0.25	0.6	8–40	83.24–92.69	0.20–2.07	This work

## Data Availability

Not applicable.

## Sample Availability

Samples of the compounds are available from the authors.
